# Mutation of the cytosolic ribosomal protein-encoding *RPS10B* gene affects shoot meristematic function in Arabidopsis

**DOI:** 10.1186/1471-2229-12-160

**Published:** 2012-09-10

**Authors:** Petra Stirnberg, Jin-Ping Liu, Sally Ward, Sarah L Kendall, Ottoline Leyser

**Affiliations:** 1 Department of Biology, University of York, Wentworth Way, York, YO10 5DD, UK; 2 Present Address: College of Agronomy, Hainan University, No. 58 Renmin Avenue, Haikou, Hainan Province, 570228, People’s Republic of China; 3 Present Address: Sainsbury Laboratory, Cambridge University, Bateman Street, Cambridge, CB2 1LR, UK; 4 Present Address: Department of Biology, Centre for Novel Agricultural Products, University of York, Wentworth Way, York, YO10 5DD, UK

**Keywords:** Shoot branching suppressor, S10e, Axillary bud, Leaf polarity, Lateral organ boundary, Auxin, Strigolactone, CUC, REV

## Abstract

**Background:**

Plant cytosolic ribosomal proteins are encoded by small gene families. Mutants affecting these genes are often viable, but show growth and developmental defects, suggesting incomplete functional redundancy within the families. Dormancy to growth transitions, such as the activation of axillary buds in the shoot, are characterised by co-ordinated upregulation of ribosomal protein genes.

**Results:**

A recessive mutation in *RPS10B,* one of three Arabidopsis genes encoding the eukaryote-specific cytoplasmic ribosomal protein S10e, was found to suppress the excessive shoot branching mutant *max2-1*. *rps10b-1* mildly affects the formation and separation of shoot lateral organs, including the shoot axillary meristems. Axillary meristem defects are enhanced when *rps10b-1* is combined with mutations in *REVOLUTA*, *AUXIN-RESISTANT1*, *PINOID* or another suppressor of *max2-1*, *FAR-RED ELONGATED HYPOCOTYL3*. In some of these double mutants, the maintenance of the primary shoot meristem is also affected. In contrast, mutation of *ALTERED MERISTEM PROGRAMME1* suppresses the *rps10b-1*axillary shoot defect. Defects in both axillary shoot formation and organ separation were enhanced by combining *rps10b-1* with *cuc3,* a mutation affecting one of three Arabidopsis NAC transcription factor genes with partially redundant roles in these processes. To assess the effect of *rps10b-1* on bud activation independently from bud formation, axillary bud outgrowth on excised cauline nodes was analysed. The outgrowth rate of untreated buds was reduced only slightly by *rps10b-1* in both wild-type and *max2-1* backgrounds. However, *rps10b-1* strongly suppressed the auxin resistant outgrowth of *max2-1* buds. A developmental phenotype of *rps10b-1*, reduced stamen number, was complemented by the cDNA of another family member, *RPS10C*, under the *RPS10B* promoter.

**Conclusions:**

*RPS10B* promotes shoot branching mainly by promoting axillary shoot development. It contributes to organ boundary formation and leaf polarity, and sustains *max2-1* bud outgrowth in the presence of auxin. These processes require the auxin response machinery and precise spatial distribution of auxin. The correct dosage of protein(s) involved in auxin-mediated patterning may be *RPS10B*-dependent. Inability of other *RPS10* gene family members to maintain fully S10e levels might cause the *rps10b-1* phenotype, as we found no evidence for unique functional specialisation of either *RPS10B* promoter or RPS10B protein.

## Background

Shoot branching exemplifies two characteristic aspects of plant development. First, the body plan is generated by the production of repetitive modules. Second, the timing of the initiation, subsequent growth, and the final morphology of these modules are flexible and responsive to internal and external cues. This second aspect suggests that plants possess mechanisms to modulate their cellular growth machinery, including complex and energy-demanding processes such as ribosomal biogenesis, cell divison and cell expansion.

During post-embryonic growth of the shoot, secondary shoot meristems can generate new growth axes. These secondary meristems include leaf-associated, branch-forming axillary meristems, and reproductive, floral meristems [1]. In many respects, these secondary meristems resemble the primary shoot meristem, which gives rise to the primary shoot axis. A common set of regulatory genes acts in their formation and patterning [2]. Few genes, such as the Arabidopsis *RAX* family [3,4] seem to function exclusively in the formation of secondary shoot meristems, possibly as position specific initiators of the shoot meristematic programme. Some of the common functions are encoded by small gene families whose members vary in their contribution with respect to meristem position, such that mutation of one family member results in a secondary shoot meristem-specific phenotype. For example*.* in Arabidopsis*,* loss of *REVOLUTA (REV)*, one of a family of five class III *HOMEODOMAIN LEUCINE ZIPPER (HDZIPIII)* transcription factor genes, leads to partial loss of axillary meristems and causes premature arrest of some floral meristems [5,6]. However, if two other family members, *PHAVOLUTA* and *PHABULOSA*, are mutated in addition to *REV*, the embryonic shoot meristem fails to form [7,8]. Similarly, within the three-member *CUP-SHAPED COTYLEDON* (*CUC*) gene family, *CUC2* and *CUC3* overlap in axillary meristem formation, while all three genes contribute to the formation of the primary shoot meristem [9-12].

Secondary shoot meristems initiate in zones where *CUC* and *HDZIPIII* expression overlap [2]. Postembryonic *CUC* expression strongly marks the boundaries of initiating lateral organs and has also been detected, at a low level, at the meristem centre [10,11,13,14]. *CUC3* for example, marks the adaxial boundary of developing leaf primordia, where secondary meristems will form [12]. *HDZIPIII* expression is initially continuous, spanning the meristem centre and the adaxial half of initiating leaves, but the leaf domain separates with its displacement from the growing meristem summit [6,15]. The abaxial side of organ primordia is marked by expression of genes from the four-member *KANADI (KAN)* family. These may limit shoot meristematic activity, because ectopic *KAN* expression abolishes shoot meristem formation, and multiple loss-of function *kan* seedlings form ectopic lateral organs [16-19]. While these and a number of other transcription factor genes are clearly involved in establishing and patterning shoot meristems, it is less clear whether and how they affect the rate of meristematic growth and organ production. For example, *HDZIPIII* family members appear to regulate the size of the central stem-cell containing zone in shoot meristems [8,20-22], and this might affect meristem activity. *CUC* expression marks zones of reduced growth within the shoot meristem [23], but also in other tissues [24].

Many of the axillary shoot meristems initiated during the lifetime of a plant cease growing after a short period, forming a small dormant bud in the leaf axil. Due to their ability to resume growth rapidly in response to an activating signal, axillary buds have been used as a model to study the regulation of meristematic activity in plants. Subtractive gene cloning in pea, and microarray analysis in Arabidopsis, show that bud activation involves a rapid, strong and coordinate upregulation of cell-cycle and protein synthesis-related genes, including many ribosomal protein (r-protein) genes, which precedes the onset of growth [25,26]. Analysis of the promoter motifs shared by these genes points to possible control by members of the TCP (TEOSINTE BRANCHED / CYCLODEA / PROLIFERATING CELL FACTORS 1 and 2) transcription factor family [26]. Of the two types of TCPs, class I is associated with growth activation and class II with growth arrest; and the DNA binding motifs identified for each class overlap partially, raising the possibility of competitive regulation via shared promoter elements [27]. In support of a role of TCPs in axillary bud growth control, loss of function of axillary shoot-meristem-specific class II TCPs, such as the *BRANCHED1 (BRC1)* and *BRC2* genes of Arabidopsis, is associated with constitutive bud activation [28,29]. The correlation between the expression of such bud-specific class II *TCP* genes and the extent of bud growth repression is generally good, but not absolute [30]. One possible explanation for this is the involvement of co-regulators of bud growth such as positively-acting TCPs.

The plant hormone auxin plays a dual role in shoot meristem growth, acting both locally along with patterning genes within the meristem, and as a long-distance signal to coordinate meristem activities within the shoot. Its patterning role has been clarified in the last decade. Transient local auxin maxima form and induce lateral organ formation in the peripheral zone of shoot meristems. These are created through directional auxin transport involving PIN1 and possibly other members of the PIN-formed protein family [31]. The protein kinase PINOID [32] is required for the observed dynamic directional changes in PIN plasma membrane localisation and auxin transport direction [33,34]. Organogenesis is thought to be induced via auxin-receptor mediated activation of members of the AUXIN RESPONSE FACTOR (ARF) transcription factor family [35], several of which are expressed at the shoot apex [36]. These might, directly or indirectly, modulate the expression of meristem patterning genes. For example, auxin-mediated repression is thought to restrict *CUC* expression to the boundaries of initiating organs [37]. In contrast, some *HD-ZIPIII* family members are auxin-induced [38].

Lateral organ development is accompanied by an inward movement of auxin through the centre of the organ primordium towards the vasculature in the subtending shoot axis [39,40]. It is thought that this triggers vascular differentiation in an interplay with the adaxial *HDZIPIII*, abaxial *KAN*, and *ARF* genes expressed within this zone [41,42], and establishes continuity with the pre-existing vasculature, in which auxin moves in a strictly basipetal (shoot-to-root) direction in the xylem parenchyma. Interestingly auxin moving in this polar transport stream (PATS) in the shoot axis has long been known to inhibit axillary shoot meristem activity in an indirect manner. These observations have been integrated into a model where both apical and axillary shoot meristem activities are governed by the ability to canalize auxin transport from developing organ primordia into pre-existing vasculature [43-45]. In addition, auxin in the PATS seems to control the production of other signals, which move root-to-shootwards in the xylem and might enter axillary shoots and regulate their growth. Auxin suppresses the biosynthesis of cytokinins [46,47], which can promote the growth of axillary buds when directly applied to them [48], and promotes the biosynthesis of the recently-discovered strigolactones [49-52], which can inhibit axillary buds upon direct application [53].

*more axillary growth2-1* (*max2-1*) is a strigolactone signalling mutant which shows constitutive axillary bud activation [54-56]. In a screen for second-site *max2-1* branching suppressors, we unexpectedly identified a mutation in *RPS10B*, one of three genes encoding protein S10e of the cytoplasmic ribosome, whose role in supporting shoot meristematic function we describe here.

## Results

### A recessive mutation in cytosolic ribosomal protein RPS10B partially suppresses *max2-1*

**Figure 1:**
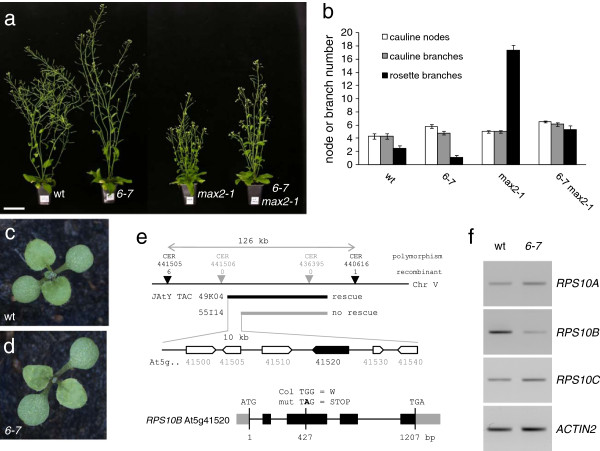
***6-7 *, a partial suppressor of *max2-1, *affects ribosomal protein gene *RPS10B. *** (**a,b**) Effect of *6-7* on shoot architecture and branching in the wild-type *MAX2* and in the *max2-1* mutant background. (**a**) Plants aged 6 weeks. Scale bar: 5 cm. (**b**) Number of cauline nodes, cauline branches and rosette branches (≥0.5 cm) at maturity (Average ± SEM, n = 10). (**c,d**) The first leaves of *6-7* mutant seedlings (**d**) are slightly more pointed than those of wild-type (**c**) seedlings. (**e**) *6-7* carries a mutation in *RPS10B*, one of three Arabidopsis genes encoding ribosomal protein S10e. Gene mapping to a 126 kb interval on chromosome 5. Population size: about 1600 mutant individuals. Mutant rescue by JAtY TAC clone 49 K04, but not by 55I14, defined six candidate genes. Only one of these, *RPS10B*, carried a nonsense mutation in its coding region. (**f**) RT-PCR analysis showing reduced *RPS10B* transcript levels in *6-7*. The primers used for RT-PCR are given in Table 5.

The strigolactone-insensitive *max2-1* mutant produces an excessive number of inflorescence branches from rosette leaf axils [54]. To identify novel regulators of shoot branching, we performed a suppressor screen in this genetic background. In one of the isolates, *6-7*, a recessive, second-site mutation, significantly reduced rosette branching. In addition, *6-7* shoots were slightly taller than *max2-1* and their primary inflorescences had a slightly higher number of cauline, leaf-bearing nodes (Figure 1a, b). We temporarily named the suppressor mutation in this line *67*. After backcrossing *6-7* to wild-type Columbia, these traits were also detected in the wild-type *MAX2* background, although the effect on branching was less striking, and could not be readily used to map the suppressor. A pointed juvenile leaf phenotype that co-segregated with the branching habit was instead used (Figure 1c, d). *67* was crossed to Landsberg-*erecta*, and the locus was mapped to a 126 kb region on chromosome 5 by assessing co-segregation of DNA polymorphisms between Landsberg and Columbia in mutant individuals from the F_2_ of this cross. JAtY TAC library clones in pYLTAC17 [57] containing large wild-type genomic inserts from the mapping interval were transformed into the mutant and assessed for rescue. This defined six candidate genes, whose coding regions were amplified from *6-7* and sequenced (Figure 1e). The sole divergence from wild type was a G to A transition, which introduced a premature termination codon in At5g41520 (*RPS10B*), one of three Arabidopsis genes encoding cytoplasmic ribosomal protein S10e. *RPS10B* transcript level was lower in *6-7* than in the wild type (Figure 1f), suggesting nonsense-mediated decay. Identity of *RPS10B* as the suppressor gene was confirmed by mutant rescue with a wild-type *RPS10B* genomic construct (Additional file 1: Table S1), and the mutant allele was named *rps10b-1.*

### *rps10b-1* affects axillary shoot initiation and growth

With wild-type Columbia plants grown in long photoperiods, floral transition is the trigger for axillary shoot initiation. The axillary shoots activate to form inflorescence branches in an apical-basal wave, i.e. from the cauline leaf axils, situated along the primary inflorescence, towards the rosette leaf axils [58]. In the wild type, only a few of the topmost rosette leaf axils produce branches, while more basal rosette axils carry arrested buds. In *max2-1*, neither the timing of axillary shoot initiation nor the outgrowth sequence is altered, but nearly all the rosette axils produce a branch [54].

**Figure 2:**
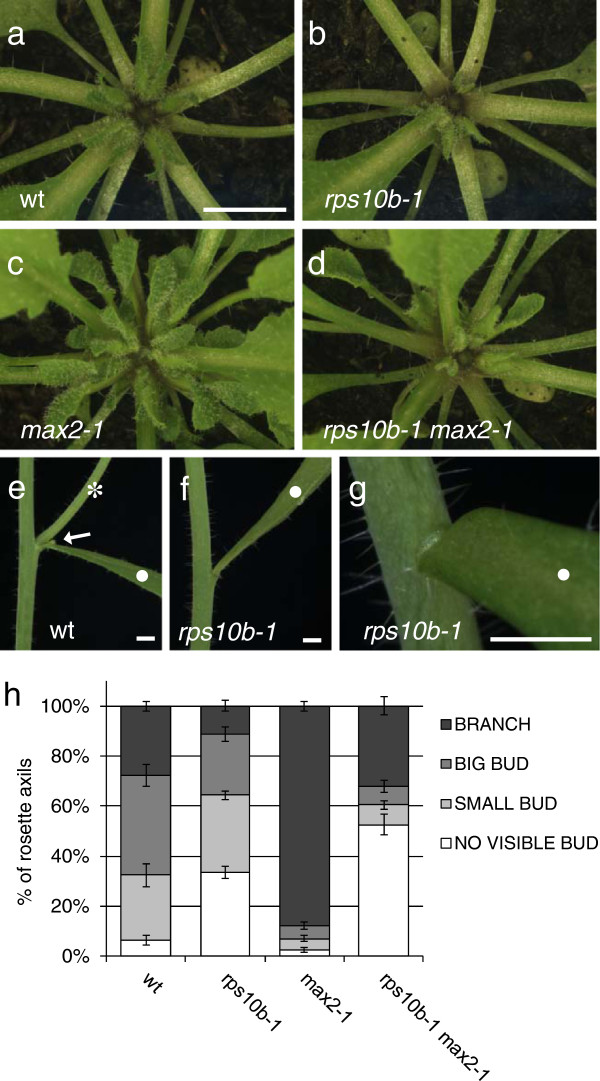
***rps10b-1 *affects axillary bud initiation and growth in the wild-type and the *max2-1 *mutant backgrounds. ** (**a-d**) Rosette centres seen from above at early flowering stage. The primary inflorescences were between 1.7 and 2 cm long and were removed to reveal the rosette leaf axils. Scale bar in (**a**) for (**a-d**) 5 mm. (**e-g**) *rps10b-1* affects axillary shoot formation at cauline nodes. Scale bars in (**e-g**) 2 mm. (**e**) Wild-type cauline node with a leaf (white dot), a cauline branch (white star) and a small accessory axillary bud (white arrow). (**f,g**) Some cauline leaf axils of *rps10b-1* appear empty. (**h**) Quantitative analysis of rosette axillary shoot development at the reproductive stage (when the tenth flower on the primary inflorescence opened). For ten rosettes per genotype, all the leaf axils were examined under a dissecting microscope and the developmental stage of the axillary shoots scored into four classes given in the key (defined in detail in the Results section). The percentages of rosette nodes occupied by each class were calculated for each individual plant and the average percentages (±SEM) for each genotype are shown.

The *rps10b-1* mutation caused a reduction in axillary shoot size at equivalent nodal positions in the rosettes of both *MAX2* and *max2-1* plants (Figure 2a–d). In addition, one or two axils at the top of the rosette often appeared to be empty. A small proportion of the *rps10b-1* cauline leaf axils were also empty (Figure 2e–g, Table 1), and remained so until maturity. This indicates that *rps10b-1* affects axillary shoot initiation. Either a delay in axillary shoot formation, or an additional effect on axillary bud growth rate, might cause the reduced size of *rps10b-1* buds.

**Table 1:** **Effect of *rps10b-1 *on cauline vegetative node development**

**Genotype**	**Axillary shoot score**			**Leaf score**			**Nodes scored**^**a**^
	**% of cauline nodes**			**% of cauline nodes**			
	**with branch**	**with bud**	**without bud**	**normal**	**leaf-stem fusion**	**without leaf**	
wild type	100.0	0.0	0.0	100.0	0.0	0.0	153
*rps10b-1*	94.4	1.7	3.9	90.5	2.2	7.3	179
*max2-1*	100.0	0.0	0.0	100.0	0.0	0.0	163
*rps10b-1 max2-1*	97.3	0.5	2.3	96.4	3.2	0.5	220

^a^The cauline vegetative nodes along the primary inflorescence of 38–40 plants per genotype were scored by the naked eye.

To quantify these phenotypes, we examined flowering plants under a dissecting microscope and assessed axillary shoot development at consecutive nodal positions throughout the rosette. Four developmental stages were defined, and the proportions of rosette axils at each stage were calculated for ten individual plants per genotype (average proportions ± SEM shown in Figure 2h). The stages were defined as follows: 1. Branches (inflorescence length above 3 mm), 2. Big axillary buds whose inflorescence had not yet significantly elongated. 3. Small buds with leaf primordia clearly visible but shorter than 2 mm. 4. Apparently empty axils lacking visible axillary leaf primordia (it was not possible at the magnification used to determine whether an axillary meristem had been initiated or not). The frequency of class 4 was negligible in both wild-type and *max2-1* rosettes, but these genotypes differed with respect to the proportions of the three more advanced classes. Compared to the wild type, *max2-1* showed a dramatic increase in the most advanced class, balanced by a decrease of the two intermediate classes. In contrast, for *rps10b-1* in both the *MAX2* and *max2-1* backgrounds, the proportion occupied by the most advanced class decreased, and this was balanced by an increase in the proportion of apparently empty axils, with little change in the intermediate classes. These results indicate that *RPS10B* promotes axillary shoot development from an early stage, including both axillary bud formation and possibly subsequent bud growth. In contrast, *MAX2* represses only the later stages of bud activity [54], suggesting that *RPS10B* acts at least in part independently of *MAX2*.

**Figure 3:**
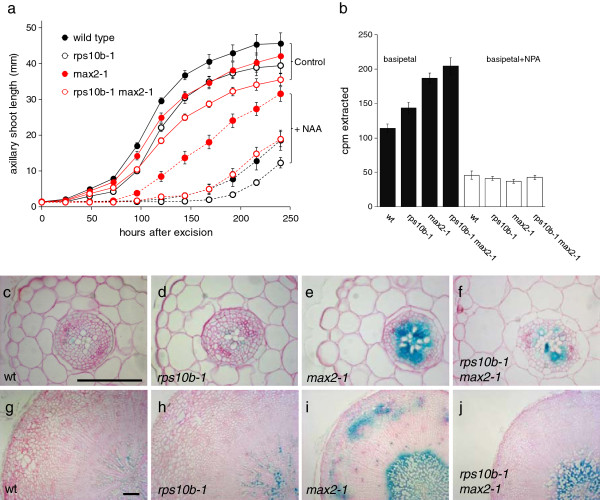
***rps10b-1 *suppresses some auxin-related phenotypes of *max2-1. *** (**a**) Growth and auxin sensitivity of cauline axillary buds in isolation from the plant. Single cauline nodes with a bud smaller than 2 mm were excised from sterile plants and inserted between two agar slabs in a Petri dish. Time course of bud elongation in the absence (solid lines, Control) and in the presence of synthetic auxin (1-naphthaleneacetic acid at 1 μM added to the apical agar slab, dashed lines, NAA). Average ± SEM, n = 12– 20. (**b**) Polar auxin transport along 1.5 cm stem segments from the base of the primary inflorescence of 6-week-old plants. 1 μM ^14^C-IAA was in contact with the apical end of the segment without (black bars) or with the polar auxin transport inhibitor naphthylphtalamic acid (NPA, at 1 μM, white bars) for 6 hours. The mean radioactivity extracted from the basal 5 mm of the segments ± SEM is shown (n = 23–24 minus NPA, n = 9-10 plus NPA). (**c-j**) Activity of the auxin response reporter DR5::GUS in hypocotyls. Hypocotyls were stained for GUS activity (blue), fixed, embedded, sectioned at 10 μm, and counterstained with ruthenium red. (**c-f**) Hypocotyl sections from 2-week-old seedlings grown in continuous light. (**g-j**) Hypocotyl sections from 9-week-old plants grown in short photoperiods. Scale bar in (**c**) for (**c-f**) and in (**g**) for (**g-j**) 100 μm.

To assess whether *rps10b-1* affects axillary shoot growth independently from initiation, we studied the outgrowth kinetics of axillary inflorescences on isolated cauline nodes. Nodal explants, consisting of a cauline axillary bud smaller than 2 mm and 5–7 mm of the primary inflorescence stem above and below the node, were inserted between two agar slabs in a Petri dish (as described in [59]). The length of the axillary buds was monitored over a 10 day period. *rps10b-1* caused a slight delay in inflorescence outgrowth in both *MAX2* and *max2-1* backgrounds (Figure 3a, solid lines).

### *rps10b-1* does not restore strigolactone responses to *max2-1*, but confers auxin-related phenotypes antagonistic to those of *max2-1*

In addition to increased branching, the *max2-1* mutant has a range of phenotypes associated with its strigolactone insensitivity. These include an elongated hypocotyl and overexpression of the strigolactone biosynthetic genes *CAROTENOID CLEAVAGE DIOXYGENASE7* (*CCD7*) and *CCD8*, which are feedback-downregulated by strigolactone signalling [52,55,56]. In a hypocotyl growth inhibition assay, *rps10b-1* did not suppress the strigolactone insensitivity of *max2-1* (Additional file 2: Figure S1). Furthermore, *rps10b-1* did not affect levels of *CCD7* or *CCD8* transcript characteristic of the *MAX2*- or *max2-1*-backgrounds (Additional file 3: Figure S2). Therefore, the suppression of *max2-1* by *rps10b-1* is specific to axillary shoot growth and does not involve a global restoration of strigolactone responsiveness.

Auxin has been implicated in both axillary meristem initiation and outgrowth. Furthermore, *max2-1,* in common with other strigolatone mutants, displays a number of auxin-related phenotypes, which led to the hypothesis that strigolactones act by restricting polar auxin transport. We therefore assessed the effect of *rps10b-1* on these auxin-related phenotypes. The outgrowth of wild-type buds is strongly delayed by apical supply of the synthetic auxin naphthalene acetic acid (NAA), but *max2-1* axillary buds are resistant to this auxin effect [55,59,60] (Figure 3a). In the wild-type background, *rps10b-1* delayed outgrowth only slightly, similar to its effect in the absence of auxin. However, in combination with *max2-1, rps10b-1* substantially delayed bud outgrowth, such that the outgrowth of double mutant buds on auxin-treated explants was nearly identical to wild-type buds. Thus, *rps10b-1* suppresses *max2-1* bud auxin resistance.

A second auxin-related phenotype of *max2-1* is an increase in basipetal transport of radiolabeled auxin through primary inflorescence stem segments [55,60]. We found that *rps10b-1* did not affect this phenotype (Figure 3b). Rather, the mutation slightly increased the amount of auxin transported in both *MAX2* and *max2-1* backgrounds.

Third, the auxin response reporter construct DR5::GUS [61] has increased activity in the main shoot axis of *max2-1* plants [60], associated with increased amounts of auxin moving in the PATS [44]. We found that this increase in DR5::GUS expression was partially suppressed in *rps10b-1 max2-1*. This effect was observed in hypocotyls from 2-week old seedlings (Figure 3c–f) as well as hypocotyls from 9-week-old short-day grown plants, which had undergone secondary thickening (Figure 3g–j). In the *MAX2* background, *rps10b-1* had little effect, with xylem-associated DR5::GUS activity possibly slightly increased. These differences in reporter activity do not simply reflect differences in bud activity, because the 2-week-old seedlings had not yet initiated axillary buds. In summary, *rps10b-1* partially rescued some of the auxin-related phenotypes of *max2-1*, indicating that *RPS10B* may act by modulating auxin responsiveness or homeostasis.

### *rps10b-1* in high-branching mutant backgrounds

**Figure 4:**
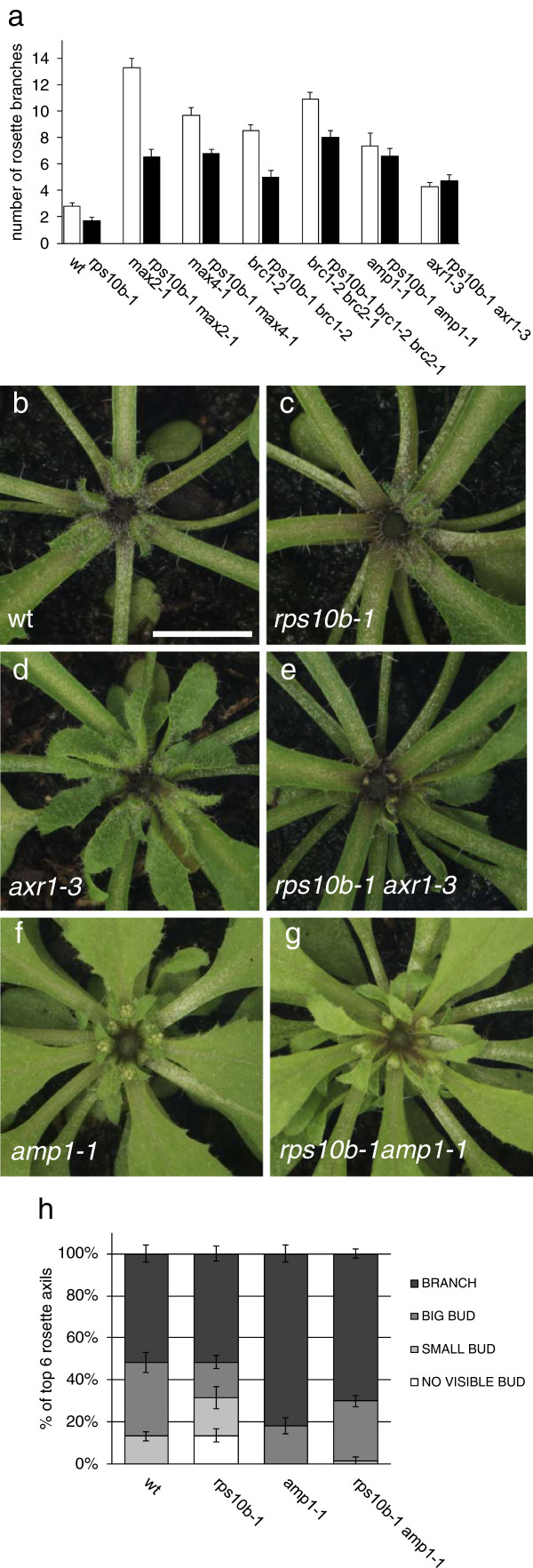
**Interaction of *rps10b-1 *with high-branching mutants. ** (**a**) Effect of *rps10b-1* on the number of rosette branches at maturity in *max2-1, max4-1*, *brc1-2, brc1-2 brc2-1*, *amp1-1* and *axr1-3* mutant backgrounds. Branches ≥ 0.5 cm were counted, average ± SEM, n = 8-10. (**b-g**) Rosette centres of selected genotypes from (**a**), seen from above at early flowering stage. The primary inflorescences were between 3.0 and 3.4 cm long and were removed to reveal the rosette leaf axils. Scale bar in (**b**) for (**b-g**) 5 mm. (**h**) The *rps10b-1* bud initiation defect at apical rosette axils is rescued in the *rps10b-1 amp1-1* double mutant. Quantitative analysis of rosette axillary shoot development at the reproductive stage was carried out as in Figure 2h, but only the topmost six rosette leaf axils of each plant were scored, n = 10-11.

To learn more about the mode of *RPS10B* action we assessed its genetic interactions with other known shoot branching regulatory genes. First, the effect of the *rps10b-1* mutation in high-branching mutant backgrounds other than *max2-1* was assessed (Figure 4a). As expected, the strigolactone biosynthetic mutant *max4-1* (*ccd8)*[62], was partially suppressed. *brc1-2* and *brc2-1* are loss of function alleles of bud-specific class II *TCP* transcription factor genes [28]. As with *max2*, excessive branching of *brc1-2* is strigolactone-insensitive [53]. *brc1-2* and the *brc1-2 brc2-1* double mutant were also partially suppressed by *rps10b-1*. In all tested double and triple mutant combinations of *rps10b-1* with *max4*, *brc1* or *brc2*, empty axils were present at apical nodes in the rosette at maturity. Thus, as with *max2*, at least part of the suppression by *rps10b-1* in these backgrounds resulted from a defective or delayed axillary shoot formation.

Perception of auxin by the TIR1/AFB auxin receptors triggers the ubiquitin-mediated degradation of Aux/IAA proteins, which are repressors of the AUXIN RESPONSE FACTOR (ARF) transcriptional regulators [63]. This degradation requires the AUXIN-RESISTANT1 (AXR1) protein [63,64]. Mutation of *AXR1* has little effect on bud initiation, but results in increased and auxin resistant bud outgrowth [65,66]. In combination with *rps10b-1*, the *axr1-3* mutant allele surprisingly enhanced the suppression of axillary bud development at both apical rosette (Figure 4b–e) and cauline nodes. In some experiments, as shown in Figure 4b–e, buds in these positions were considerably smaller than those of either single mutant. In other experiments, a large proportion of cauline and apical rosette axils appeared completely empty in *rps10b-1 axr1-3* plants. In addition, the primary inflorescence meristem of *rps10b-1 axr1-3* plants frequently aborted. Between 20% and 50% of the double mutant individuals, but neither of the single mutants, had this phenotype. These observations suggest that *RPS10B* and *AXR1* interact to promote shoot meristem development. However, this interaction appeared to be positionally restricted. At more basal rosette nodes of double mutant plants, bud behaviour resembled the *axr1*single mutant; axillary buds initiated and formed inflorescence branches, such that rosette branch numbers of *axr1-3* and *rps10b-1 axr1-3* at maturity did not differ significantly (Figure 4a).

Mutation of *AMP1*, which encodes a putative carboxypeptidase with unknown molecular function, causes a range of phenotypes related to shoot meristem function including constitutive axillary bud activation, increased shoot meristem size, increased rate of leaf initiation, and increased cytokinin content [67-70]. The defective axillary bud formation in apical rosette nodes typical of *rps10b-1* was completely suppressed in an *amp1-1* background (Figure 4b, c, f–h), and at maturity, the average branch number of *rps10b-1 amp1-1* plants did not differ significantly from *amp1-1* plants. Genetic analysis by Vidaurre and coworkers [71] suggests a major function of ARF-mediated auxin signalling in embryogenic shoot meristem formation and vascularisation might be the downregulation of AMP1 activity. In the light of this finding, the genetic interaction with *amp1-1* further supports the idea that reduced ARF-mediated auxin signalling is involved in the *rps10b-1* meristematic phenotypes.

### *rps10b-1* in low-branching mutant backgrounds

**Figure 5:**
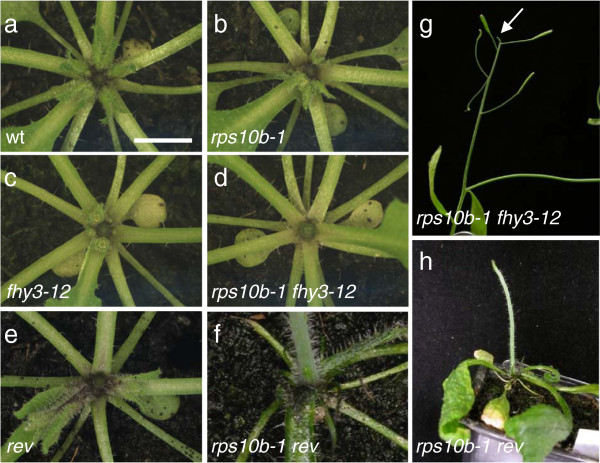
**Interaction of *rps10b-1 *with the low-branching mutants *fhy3-12 *and *rev. *** (**a-f**) Rosette centres of wild-type, single and double mutant plants at early flowering stage. Except for *rps10b-1 rev* (SALK_102345), the primary inflorescences were removed to reveal the rosette leaf axils. Note the complete absence of rosette axillary buds in *rps10b-1 fhy3-12* and *rps10b-1 rev*, and the filament-like lateral organs at the top of the *rps10b-1 rev* rosette which are likely the youngest, radialised leaves. (**g,h**) Other shoot-meristem-related phenotypes of double mutant plants. **(g)** Abortion of the primary inflorescence meristem of an *rps10b-1 fhy3-12* plant in a short, pin-like structure (arrow). (**h**) *rps10b-1 rev* primary inflorescences were pin-like and lacked lateral organs.

We also analysed the effect of *rps10b-1* in genetic backgrounds characterised by reduced branching. First, we constructed a double mutant of *rps10b-1* with another non-allelic *max2-1* suppressor from our screen, *far-red elongated hypocotyl3-12* (*fhy3-12)*. This is a loss-of function allele of the transcriptional activator *FHY3*[72]. This mutation suppresses *max2-1* by reducing bud activation, with negligible effects on axillary shoot formation; and our data suggest that auxin might be central to its branching phenotype [73]. *rps10b-1 fhy3-12* double mutant plants showed a near-complete loss of rosette axillary buds (Figure 5a–d). Furthermore, the primary inflorescence meristem of double mutant plants often aborted during the reproductive phase, a phenotype not observed with either single mutant (Figure 5g). The frequency of abortion ranged from 30% to 90% in different experiments.

**Figure 6:**
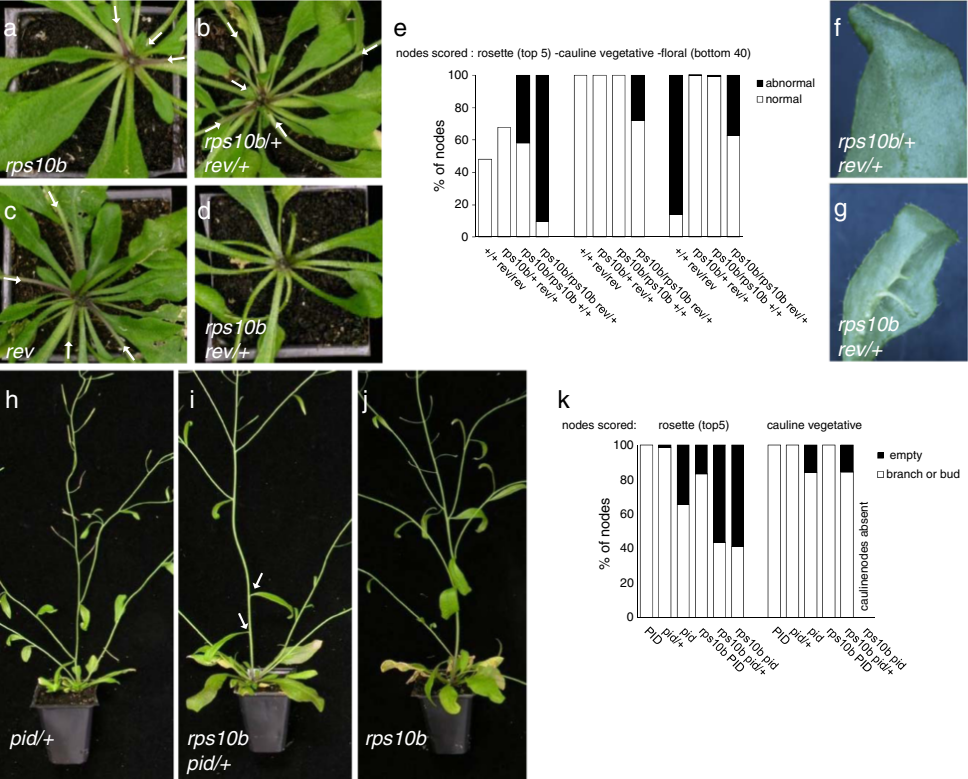
**Dosage effects of *REV *and *PID *in the *rps10b-1 *mutant background. ** (**a-g**) F_2_ from a cross of *rps10b-1* with the *rev* mutant. (**a-d**) Rosettes of F_2_ plants genotyped for *RPS10B* and *REV*. The primary inflorescences were removed. White arrows mark axillary shoots (branches or axillary buds). In the presence of functional *RPS10B* (one copy in **b**, two copies in **c**), loss of one (**b**) or both (**c**) functional *REV* copies does not noticeably affect rosette branching. In the *rps10b* mutant background (**a,d**) loss of one functional *REV* copy (**d**) completely abolishes axillary bud formation. (**e**) Quantification of axillary shoot development of genotyped F_2_ segregants. The proportions of rosette, cauline and floral nodes showing normal versus abnormal axillary shoot development are plotted. For vegetative nodes, development was classified as abnormal if the axil appeared empty. For floral nodes, development was classified abnormal when the node was occupied by a pedicel- or filament-like structure, and normal when it carried a flower or silique. For the rosette nodes of *rev* and *rps10b/+ rev/+* plants only the number of branches was scored, thus the white bar represents a minimum estimate of the proportion of normal nodes and the proportion of abnormal nodes is not given. 7 to 21 individuals were scored per genotypic class. (**f,g**) Some leaves of *rps10b rev/+* plants had outgrowths from the midvein at the abaxial side. *rps10b/+ rev/+* leaves appeared normal. (**h-k**) F_3_ from a cross of *rps10b-1* with the *pid-14* T-DNA insertion allele (SALK_049736). A *rps10b pid-14/+* segregant with two basal nodes lacking axillary shoots (**i**, white arrows), occurrence of this phenotype in *pid-14/+* controls (**h**) was negligible and in *rps10b* controls (**j**) less frequent (see **k**). (**k**) Quantification of cauline and rosette axillary shoot development of genotyped F_3_ segregants. Analysis was done as in (**e**). 7 to 16 individuals were scored per genotypic class.

As described earlier, mutation of the *HDZIPIII* gene *REV* causes partial defects in axillary meristem formation and floral meristem maintenance. In addition, the *HDZIPIII* family members redundantly specify adaxial leaf identity, but *rev* loss-of-function mutant leaves appear normal [7,8]. We generated double mutants between *rps10b-1* and a *rev* T-DNA insertion allele, SALK_102345 (Figure 5f, h). These were highly abnormal. Successive leaves became increasingly needle-like, and axillary shoots were absent. The primary stem was short, pin-like and lacked flowers. Thus, *rps10b-1* strongly enhanced the loss of *REV* function with respect to both leaf polarity and axillary shoot formation. The F_2_ analysis also revealed that a single copy of the *rev* mutant allele strongly enhanced the axillary shoot phenotypes in the *rps10b-1* mutant background, while *rps10b-1/+ rev/+* axillary shoot development was normal (Figure 6a–e*). rps10b-1 rev/+* plants had normal stature and slight defects in floral meristem maintenance. Their leaf polarity appeared largely normal, except that a few leaves had reduced lamina, from which the midvein separated as an abaxial outgrowth at the distal end of the leaf (Figure 6f, g). The strongest effect of *REV* haploinsufficiency concerned axillary shoot formation. Nearly all the rosette and a substantial proportion of cauline leaf axils were empty (Figure 6d, e). This demonstrates a dosage dependence of *REV* in the *rps10b-1* background, which is not seen in the wild-type *RPS10B* background, where *rev* appeared recessive.

The *PID* protein kinase is required for dynamic changes in plasma-membrane localisation of PIN auxin transporters and thereby auxin transport direction [32-34,74]. Plants homozygous for strong *pid* mutant alleles are defective in flower formation, and the few flowers produced are abnormal and sterile [75]. We crossed *rps10b-1* with a *pid-14* heterozygote (SALK_049736 [34,76]) and homozygous double mutants were identified in segregating *rps10b-1 pid-14/+* F_3_ families (Figure 6h–k). In addition to the defect in flower formation, which has been described, homozygous *pid-14* segregants from *RPS10B pid-14/+* control F_3_ families showed mild defects in cauline and axillary bud initiation similar to the *rps10b-1* single mutant. *pid-14* heterozygotes from the control F_3_ were indistinguishable from *PID* segregants and wild-type controls. The double mutants segregating in the progeny of *rps10b-1 pid-14/+* plants had a more severe phenotype than *pid-14* alone, as neither cauline leaves nor branches, nor flowers were produced on the primary inflorescence, and the proportion of empty rosette axils was increased. Furthermore *rps10b-1 pid-14/+* F_3_ individuals also showed slightly enhanced axillary shoot defects when compared with *rps10b-1 PID* F_3_ segregants or *rps10b-1* controls. The proportions of empty cauline and rosette axils were increased (Figure 6k). Although less striking than with *REV*, there is a *PID* dosage effect in the *rps10b-1* background, demonstrating that partial loss of this r-protein increases sensitivity to reduced function of both PID and REV.

### *RPS10B* supports *CUC* gene function

**Figure 7:**
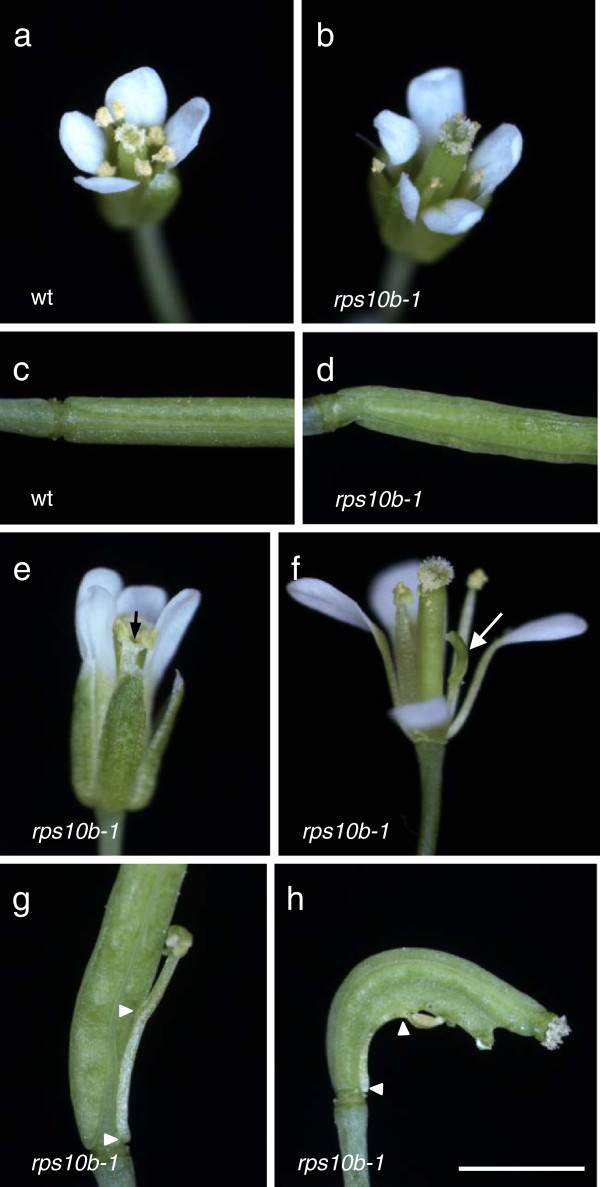
***rps10b-1 *floral organ phenotypes. ** (**a-d**) Increased variation in floral organ number. Wild-type flower with four (**a**)**,***rps10b-1* flower with five petals (**b**). Developing siliques of wild type (**c**) with two carpels**,** of *rps10b-1* (**d**) with three carpels. (**e,g,h**) Defective organ separation. Fusion of two stamen filaments indicated by black arrow in (**e**). Fusion of stamen filaments to the gynoeceum marked by arrowheads in (**g,h**). (**f,g,h**) Mis-specification of organ identity. Stamens in (**f**, white arrow), (**g**) and (**h**) showing carpelloid features. Scale bar in (**h**) for (**a-h**): 2 mm.

As described above, *rps10b-1*caused failure of the primary shoot meristem or of floral meristems in some mutant backgrounds. This could point to a more general role of *RPS10B* in supporting shoot meristematic function, which is also indicated by other weakly penetrant traits observed with the *rps10b-1* single mutant. In *rps10b-1* flowers, the number, identity and separation of lateral organs were affected (Figure 7). Sepal, petal, stamen and carpel numbers were more variable than in the wild type (Table 2). A substantial proportion of *rps10b-1* flowers lacked one stamen, while petal and carpel numbers were more often increased than decreased (Figure 7a–d). Fusion between organs in one whorl was sometimes detected, most frequently for the stamens (Figure 7e). Furthermore, some stamens were green and possibly carpelloid (Figure 7f) and/or were partly fused to the gynoeceum (Figure 7g–h).

**Table 2:** **Lateral organ numbers of wild-type and *rps10b-1 *flowers **^**a**^

**Genotype**	**Sepal**		**Petal**		**Stamen**		**Carpel**	
	**Mean ± SEM**	**Range**	**Mean ± SEM**	**Range**	**Mean ± SEM**	**Range**	**Mean ± SEM**	**Range**
wild type	4.00 ± 0.00	4	4.00 ± 0.00	4	5.92 ± 0.03	5-6	2.00 ± 0.00	2
*rps10b-1*	4.00 ± 0.05	2-5	4.22 ± 0.06	3-6	4.17 ± 0.09	2-6	2.13 ± 0.04	2-4

^a^98-100 flowers per genotype were examined under a dissecting microscope.

**Figure 8:**
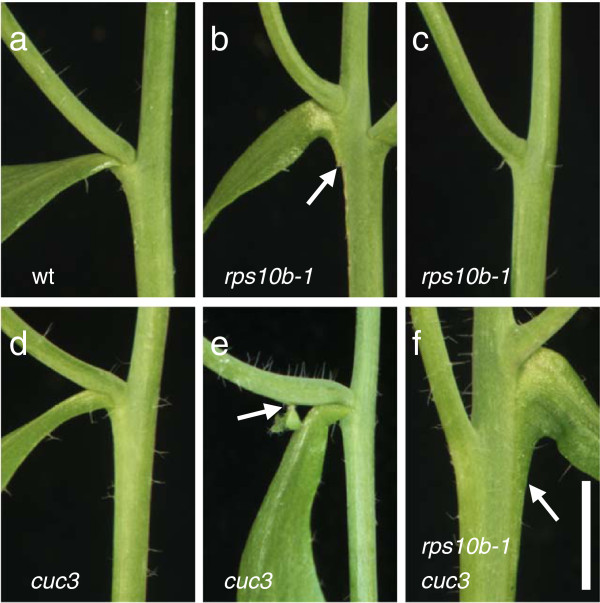
**Genetic interaction between *RPS10B *and *CUC3 *in organ separation.** (**a-f**) Cauline vegetative nodes. *cuc3* (GABI-KAT GK_302G09) nodes are phenotypically wild-type (compare **a** and **d**), with rare exceptions, as in **e** where a *cuc3* accessory axillary bud (arrow) appeared to be fused with an axillary branch. *rps10b-1* nodes occasionally show leaf-to-stem fusion (arrow in **b**) or the cauline leaf is missing (**c**), in addition to the lack of the axillary bud which was shown in Figure 2f,g. The upper of the two *rps10b-1 cuc3* double mutant nodes in **f** shows leaf-to-stem fusion (arrow). The bottom node lacks the cauline leaf and the bottom of the axillary branch may be fused with the primary inflorescence.Scale bar in (**f**) for (**a-f**): 5 mm.

Furthermore, patterning defects in addition to the lack of axillary shoots were observed at cauline nodes at low frequencies (Figure 8a–c, Table 1). The topmost cauline branches of *rps10b-1* were occasionally not subtended by a cauline leaf (Figure 8c), and fusion of cauline leaf lamina to the inflorescence stem was sometimes detected (Figure 8).

Such phenotypes suggest a role of *RPS10B* in lateral organ partitioning and separation. To test this hypothesis, we studied the genetic interaction between *RPS10B* with *CUC3*, one of three NAC transcription factor family members with partially overlapping roles in organ boundary formation. An *rps10b-1 cuc3* double mutant was constructed using a T-DNA knockout allele of *cuc3* (GABI-KAT line GK_302G09 [77]). With respect to cauline node development (Table 3 and Figure 8a, d, e), *cuc3* was nearly indistinguishable from wild type, consistent with previous reports, demonstrating redundancy in the CUC family for cauline node patterning [11,12]. Very rarely, we observed that accessory axillary shoots, which are often formed at Arabidopsis cauline nodes between the axillary branch and its subtending leaf (Figure 2e), were fused with the stem of the axillary branch (Figure 8e), or that a branch was slightly fused with the base of its subtending cauline leaf. In contrast, in the *rps10b-1 cuc3* double mutant, the frequency of obvious cauline node patterning defects was greatly enhanced. There was further loss of either the leaf or the axillary shoot, and increased fusion of organs, such that 76% of the double mutant nodes appeared abnormal (Table 3). The increase in the proportion of nodes showing abnormal leaf development (leaf absent or fused to the stem) in the double mutant, compared with *rps10b-1* alone, was highly significant (χ^2^ = 113.1, p < 0.0001). This was also the case when the proportions of nodes lacking an axillary shoot were compared (χ^2^ = 72.3, p < 0.0001).

**Table 3:** ***cuc3 ***^**a **^**strongly enhances the effect of *rps10b-1 *on the development of cauline vegetative nodes**

**Genotype**	**Axillary shoot score**			**Leaf score**			**Nodes scored**^**b**^
	**% of cauline nodes**			**% of cauline nodes**			
	**normal**	**branch-stem fusion**	**without axillary bud**	**normal**	**leaf-stem fusion**	**without leaf**	
wild type	100.0	0.0	0.0	99.4	0.6	0.0	171
*rps10b-1*	95.5	0.0	4.5	96.3	0.8	2.9	244
*cuc3*	99.2	0.8	0.0	100.0	0.0	0.0	121
*rps10b-1 cuc3*	57.6	4.6	37.7	51.7	37.7	10.6	151

^a^A T-DNA insertion allele of *cuc3*, GABI-KAT line GK_302G09, was used in this study.

^b^The cauline vegetative nodes along the primary inflorescence of 38–40 plants per genotype were scored by the naked eye.

Loss of *CUC3* function has been reported mildly to affect embryonic shoot patterning, with *cuc3* seedlings falling into two major classes: phenotypically normal, or showing one-sided cotyledon fusion. Occurrence of the severe cup-shaped phenotype caused by two-sided cotyledon fusion is rare [10,11]. This was also true for the *cuc3* allele we used (Table 4). *rps10b-1*single mutant seedlings did not show cotyledon fusion but rarely, an extra cotyledon was present. Combining *rps10b-1* and *cuc3* doubled the proportion of seedlings showing cotyledon fusion (13.9%, compared to 7.7% for *cuc3* alone, χ^2^ = 6.77, p = 0.01). It also increased the proportion of seedlings showing severe, two-sided cotyledon fusion, but not significantly (Fisher’s exact test, p = 0.06). Thus, the patterning of cotyledonary nodes appeared less sensitive to combined loss of *RPS10B* and *CUC3* function than the patterning of cauline nodes. Our observations suggest that *CUC* gene-mediated patterning depends on full *RPS10B* function, but also that this dependence varies with the developmental context.

**Table 4:** ***rps10b-1 *weakly enhances *cuc3 ***^**a **^**seedling phenotypes**

**Genotype**	**Seedlings scored**	**Normal**	**3 cotyledons**	**Cotyledons fused**		**% Abnormal**
				**One-sided**	**Two-sided**	
wild type	317	316	0	1	0	0.3
*rps10b-1*	300	299	1	0	0	0.3
*cuc3*	353	326	0	26	1	7.7
*rps10b-1 cuc3*	310	267	0	37	6	13.9

^a^A T-DNA insertion allele of *cuc3*, GABI-KAT line GK_302G09, was used in this study.

### Functional redundancy of RPS10B and RPS10C in the control of development

**Figure 9:**
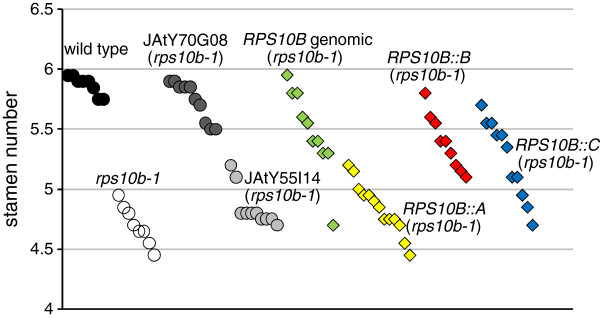
**Assessing functional redundancy of RPS10 proteins. ***RPS10B * promoter::*RPS10 *cDNA fusions were constructed for the *RPS10* family members *A*, *B* and *C* and transformed into *rps10b-1*. The T_1_ was scored for rescue of reduced stamen number, an *rps10b-1* developmental phenotype. Controls: wild type, *rps10b-1*, transgenic T_2_ plants from transformation of *rps10b-1* with JAtY TAC clone 70 G08 which spanned, and clone 55I14 which lacked the *RPS10B* genomic region; and T_1_ plants from the transformation of *rps10b-1* with a genomic *RPS10B* clone. Each symbol represents the mean stamen number of 20 flowers from the primary inflorescence of one individual plant.

Arabidopsis r-proteins are encoded by small gene families [78]. Two additional *RPS10* family members, *RPS10A* (At4g25740) and *RPS10C* (At5g52650), show 78% and 74% amino acid identity with *RPS10B*. RT-PCR from cDNA produced from total RNA of different wild-type tissues showed that all three genes are transcribed and that their relative contributions to transcript level appear invariant for the tissues we analysed (Additional file 4: Figure S3). The AtProteome database [79] points to RPS10B as the most abundant protein isoform. To test for redundancy of protein function, we amplified a cDNA corresponding to the longest annotated protein version for each member, and expressed it under the control of the *RPS10B* promoter in *rps10b-1* plants. As controls we used the wild type, *rps10b-1*, T_1_ plants from transformation of the mutant with the genomic *RPS10B* construct, and T_2_ plants from transformation of the mutant with two JAtY TAC clones, only one of which contained the *RPS10B* genomic region. Complementation efficiency was scored by counting the stamens of 20 flowers from 8–13 individual plants per genotype or construct (Figure 9). The mean individual stamen numbers ranged between 5.7 and 6 for wild-type plants; but were below 5.4 for the mutant or transformants with the JAtY TAC clone that lacked *RPS10B*. For the T_2_ transformed with the JAtY TAC containing *RPS10B*, and for 9 out of 10 T_1_ transformed with the genomic *RPS10B* construct, stamen numbers ranged from 5.4 up to the maximum values obtained for wild-type plants. A mean stamen number lower than wild type but still above those of mutant plants may be explained by a lower dose of functional *RPS10B* in some transformants than in wild type, as the majority of the JAtY T_2_ and most of the T_1_ are expected to contain one transgene copy. Of the three *RPS10B* promoter::cDNA fusions, *RPS10B::B* rescued most efficiently, however with a further reduction compared to the genomic construct, which could indicate a requirement to generate alternative transcripts, or for intronic or untranslated sequences for the proper control of *RPS10B* gene expression. The *RPS10B::C* construct complemented the stamen phenotype in about half of the T_1_; however, none of the *RPS10B::A* T_1_ was rescued. While the reason for the non-complementation by *RPS10A* is not clear, the rescue by the *RPS10C* cDNA argues against a specialised role of RPS10B within the S10e protein family.

## Discussion

### The *RPS10* gene family

*RPS10B* belongs to the three-member Arabidopsis gene family encoding the eukaryote-specific protein S10e of the small cytoplasmic ribosomal subunit [78,80]. Like most of the r-proteins, S10e is essential for the biogenesis of its ribosomal subunit [81]. It is positioned at the “beak” of the small subunit, a structure that is formed from protein and rRNA in eukaryotes, but exclusively from rRNA in bacteria [82]. The role of S10e in translation is unknown. Crosslinking experiments indicate that S10e might participate in the interaction of the small subunit with eukaryotic initiation factor 3, which functions in translation initiation [83,84].

The Arabidopsis *rps10b-1* mutant allele is transcribed and can encode a truncated protein; its recessive inheritance is consistent with either reduced or abolished protein function. A knockout allele could not be obtained from T-DNA mutant collections. The fact that cDNAs of *RPS10B* and *RPS10C* (driven by the *RPS10B* promoter) rescued an *rps10b-1* mutant phenotype to a similar extent, suggests that the RPS10B protein has not functionally diverged from other family members. We detected transcripts of all *RPS10* family members in all tissues tested, with highest transcript levels in young, growing tissues, including axillary buds (Additional file 4: Figure S3).

### The specificity of ribosomal protein mutant phenotypes

An increasing collection of ribosomal protein mutants have been recovered from screens for developmental phenotypes in Arabidopsis, with substantial overlap in the suite of phenotypes conferred by these mutations. The phenotypes include altered leaf shape (the first leaves are narrow and pointed) and the ability to enhance the phenotype of mutations that affect leaf adaxial identity, for example *asymmetric leaves1* (*as1*) or *as2*[85-91]. However, these r-protein mutations differ substantially in their effects on plant growth, which could reflect variation in the degree of genetic redundancy. In *rps10b-1*, expression of the pointed first leaf phenotype was mild*.* Leaf polarity was affected in double mutant combination with *rev* (Figures 6, 7) and we confirmed that this was also the case with *as1* (Additional file 5: Figure S4). Although we observed weak effects on growth rate, for example in axillary buds on isolated nodal segments, the shoot or organ size of mature plants was not noticeably reduced, arguing against a general growth defect. The basis of the developmental defects of r-protein mutants is unclear. Two possibilities seem likely.

First, defective ribosomes may trigger specific developmental defects through their participation in surveillance mechanisms at cell cycle checkpoints. For example in humans, redundancy of r-proteins is less common, and haploinsufficiency of S10e and several other proteins of the large or small ribosomal subunit cause Diamond-Blackfan anemia, a syndrome of specific developmental defects including the failure of red blood cell progenitors [92,93]. According to current understanding of the disease, these mutations perturb ribosome biogenesis via an imbalance in ribosome constituent stoichiometry. This is likely to increase the level of unincorporated r-proteins, several of which can bind and inactivate a ubiquitin ligase which targets the p53 tumor suppressor protein [94,95], and its resulting stabilization triggers cell cycle arrest in red blood cell progenitors. It may be that this surveillance mechanism operates in certain cell types only, for example cells that proliferate very rapidly [96,97], which could explain the developmental specificity of the phenotype. It is not known whether similar surveillance systems operate in plants.

Second, ribosome insufficiency, the production of disfunctional ribosomes, or the lack of ribosomes containing a specific r-protein variant could affect the production of specific proteins more than others. For example developmental patterning or cell cycle genes might crucially depend on particularly high translation rates or on a specialized ribosome variant. An interesting case here is the Aux/IAA transcriptional repressors, which are central to auxin-regulated gene expression. Some members of this protein family have extremely short half-lives, in the order of 5 minutes [98], and are maintained at steady state level in cells with a particular auxin concentration. Upon auxin addition, their half-lives are further reduced [64,99], resulting in their depletion and hence the up-regulation of transcription by a sub-family of ARFs. Because of the need for continuous replenishment of these proteins, it is possible that developmental events dependent on dynamic changes in auxin signaling are particularly sensitive to inefficient ribosomes. Alternatively, the consequences of reduced or altered ribosome function might be enhanced by specific features of the mRNA encoding a protein, for example by the presence of upstream ORFs, which require translation re-initiation. This is the case for the mRNAs of several ARF transcription factors, including ARF3 (ETTIN) and ARF5 (MONOPTEROS, MP), and was proposed to cause *arf*-like developmental phenotypes of the r-protein mutant *short valve1* (*rpl24b*) [100]. Another ribosome-dependent process, which might potentially be affected is miRNA-directed translational regulation [101,102]. Many of the genes involved in meristem patterning and adaxial identity are regulated by small RNAs [103,104].

The work presented here is suggestive of this second set of possibilities, because many of the effects we observe are indicative of a general lack of robustness of the adaxial patterning system, with the *rps10b-1* mutation rendering the system sensitive to the dosage of other important regulatory components.

### *RPS10B* and shoot meristem function

Despite the intuitive lack of specificity expected from a ribosomal protein mutation, it is clear that mutation of *RPS10B* causes a syndrome of phenotypes that can be attributed to patterning events at the shoot apical meristem, and particularly to the establishment of boundaries between the meristem and the leaf, and to a lesser extent, within the leaf.

*rps10b-1* suppresses excessive shoot branching in the *max2-1* mutant background. A reduced ability to initiate or maintain axillary shoot meristems is a major cause of this suppression. The axillary shoot defects of *rps10b-1* were enhanced in double mutant combination with *axr1*, *fhy3*, *rev*, and *pid*, and were sensitive to reductions in the dose of *REV* and *PID*. Moreover, maintenance of the primary shoot apical meristem was partially affected in combination with *axr1* and *fhy3*; a phenotype not observed in the single mutants. Finally, *rps10b-1* enhanced the floral meristem defects of *rev* and *pid*. This indicates a general role of *RPS10B* in shoot meristem function. In addition, the *rps10b-1 rev* double mutant phenotype revealed that *RPS10B* is involved in leaf polarity, like many other r-protein genes.

While axillary meristem defects have not yet been reported for r-protein gene single mutants (perhaps because they are relatively weak), introgression of *piggy1* (*rpl10ab*) into a *rev* mutant, *stv1* (*rpl24b*) into an *arf3* mutant and *rpl4d* into an *as1* mutant background resulted in striking axillary and/or floral meristem defects [88,100,105]. Formation of the embryonic shoot meristem; and shoot meristem, vascular and leaf patterning crucially depend on an interaction between *HDZIPIII* and *KAN* genes [2,19,106]. Furthermore, axillary and embryonic shoot meristem formation are similar in many respects and likely to share the *HDZIPIII /KAN* patterning mechanism. With respect to leaf patterning, r-protein genes were found to promote genetically the adaxialising role of the *HDZIPIII* genes, and to antagonise the action of the abaxialising *KAN* genes [105,107]. *RPS10B* genetically promoted the action of *REV* both in shoot meristem function and leaf polarity. Interestingly, axillary meristem formation appeared more sensitive to halving the *REV* dose in the *rps10b-1* background, than did leaf polarity. This supports the notion that *RPS10B* acts at least partly via meristem establishment itself, and not only via the specification of leaf adaxial fate, which is a prerequisite for axillary meristem initiation in Arabidopsis [5,7,8,16]. Despite the strong genetic interactions between r-protein genes and the *HDZIPIII/KAN* pathway, further analysis did not implicate any of the ad- or abaxial polarity genes examined as direct targets of ribosomal regulation [88,91,105,107].

The *rps10b-1* mutant displayed other shoot meristem-related phenotypes that were not enhanced in combination with *rev*. Cauline nodes lacked a leaf, or the leaf was rudimentary. Sometimes, the cauline leaf margin was fused to the stem. Floral organ numbers were more variable than in the wild type and, organ fusion within and between whorls occurred. Such phenotypes indicate misregulated organ separation. Some *rps10b-1* phenotypes resemble loss of function, while others resemble gain of function phenotypes described for the three partially redundant *CUC* genes [9-12,108-110]. Furthermore, combining *rps10b-*1 and *cuc3* enhanced organ separation defects which were rare in the single mutants, most dramatically at cauline nodes. In the r-protein mutant *rpl27ac-1d*, *CUC2* was mislocalised during embryonic shoot meristem formation [91]. Interestingly, the leaf polarity regulators *AS1* and *AS2* have been implicated in *CUC* gene regulation and organ boundary formation [111-113]. Conversely, mutant phenotypes suggest a role for *CUC* genes in leaf polarity [11]. This suggests that the well-known ribosomal regulation of leaf polarity, and the organ boundary role we describe here for *RPS10B* could have a shared molecular basis.

### *RPS10B* and auxin

A common feature of many of the developmental events described above is their dependence on, or interaction with auxin and its directed transport. The formation of both the leaf-meristem boundary and the leaf abaxial-adaxial boundary involve the specific and dynamic reorientation of auxin transport paths and hence auxin distribution patterns [14]. Consistent with the importance of auxin in these events, the general reduction in the robustness of the patterning of these boundaries in the *rps10b-1* mutant is associated with a range of auxin-related phenotypes.

First, mutations affecting auxin signalling or transport enhanced some of these defects. The auxin signalling mutation *axr1-3*, which does not affect axillary shoot formation in a wild-type background [66], enhanced axillary bud loss in combination with *rps10b-1*. In addition, a novel phenotype of primary inflorescence meristem arrest was displayed by some *axr1-3 rps10b-1* double mutant plants. The effects of reduced or abolished function of the auxin transport regulator *PID* on lateral organ formation were enhanced in the *rps10b-1* mutant background. The effect of both these auxin-related mutations may be to interfere with *ARF*–regulated developmental programmes either globally (*axr1*) or through altered auxin distribution (*pid*). Mutation of the transcriptional activator *FHY3*, another *max2* branching suppressor from our screen, very strongly enhanced axillary meristem failure when combined with *rps10b-1*, and also caused inflorescence meristem arrest. We hypothesise that *FHY3* also regulates branching via auxin signalling or homeostasis [73].

Second, the *amp1-1* mutation suppressed the axillary meristem failure of *rps10b-1* in the double mutant. Although the molecular function of AMP1 is not known, loss-of-function mutant phenotypes suggest that it restricts shoot meristematic growth [70,114]. Increased levels of cytokinins have been detected in *amp1* plants [67,68], which might cause their increased meristematic stem cell activity [115]. Interestingly, a link between *AMP1* and *ARF*-mediated auxin signaling has recently been proposed. *amp1* suppresses the effect of loss of *ARF5* (*MP*) in embryonic shoot meristem development and vascularisation, indicating that one important activity of *MP* might be to antagonise *AMP1*[71]. In this way, auxin signalling in the shoot meristem could sustain the stem cell pool required for future lateral organ formation. The genetic interactions of *rps10b* with *axr1*, *pid*, and *amp1* are consistent with *RPS10B* supporting stem cell production indirectly by maintaining *ARF*-mediated auxin signalling.

### RPS10B and axillary bud outgrowth

In addition to axillary meristem specification defects, which likely underlie the poor axillary shoot formation phenotype of the *rps10b-1* mutant, we also detected defects in axillary meristem activity, which may contribute to the suppression of shoot branching in *max2*. Because of the effects on axillary bud formation, it was difficult to ascertain the effect of *rps10b* on bud outgrowth in intact plants. Therefore, we used excised cauline nodes, which were selected for approximately equal bud size at the start of the experiment. Except for one specific situation, which is discussed below, the effect of *rps10b* on bud outgrowth rate was surprisingly small, given the transcriptional evidence for high r-protein synthesis in active buds [25,26]. This could indicate that loss of *RPS10B* was compensated by functional family members. Mechanisms that ensure that ribosomal components are produced in stochiometric amounts are better studied in other organisms, but they are likely to operate in plants as well [116,117]. We detected at most a slight upregulation of *RPS10A* or *C* transcripts in *rps10b* by semi-quantitative RT-PCR (Figure 1f). However, the example of the Arabidopsis *rpl4a* and *rpl4d* mutants shows that compensation at the protein level can occur in the absence of detectable compensation at the transcript level [118].

The F-box protein MAX2 is required for normal strigolactone responsiveness, and is thought to act in an E3 ubiquitin ligase, selecting unknown protein targets for degradation [119,120]. Strigolactones are negative regulators of PIN protein levels, and of polar auxin transport in the vasculature [55]. Recent studies with excised axillary buds, to which a synthetic strigolactone was applied via the basal internode yielded two interesting observations. First, the ability of strigolactone to inhibit single excised buds required apical auxin; second, if buds on two consecutive nodes were excised, basal strigolactone enhanced the growth differential or competition between them, rather than inhibiting both [55,121]. This fits with a model of bud regulation via auxin transport canalisation, where bud activation requires the export of auxin via a shared auxin transport route in the stem, and strigolactones inhibit this process by restricting PIN protein accumulation [45]. Consistent with this, axillary buds of strigolactone mutants, including *max2*, are moderately resistant to apically applied auxin [55,60]. Interestingly, we found that the growth-inhibiting effect of *rps10b* on auxin-treated *max2* buds was much stronger than for other genotypes and treatment combinations, such that bud outgrowth kinetics of the auxin-treated double mutant were restored to wild-type. This could indicate that *rps10b* specifically suppresses a downstream effect of the *max2* mutation in bud outgrowth. This effect might be auxin-related, as *rps10b* specifically suppressed auxin responsive gene expression, as reported by DR5::GUS activity, in the shoot axis of *rps10b max2*, while it did not have this effect in the *MAX2* background. A mode of action different from strigolactone / MAX2 is suggested by the fact that *rps10b* did not antagonise the effect of *max2* on stem polar auxin transport; and the fact that *rps10b* did not restore the altered shoot vascular architecture of *max2* back to wild type (compare sections of older plants in Figure 3g-j). The vasculature of *max2* stems shows increased activity of the PIN1::PIN1-GFP reporter [55,60]. In a recent evaluation of the vascular role of the *HDZIPIII* and *KAN* genes, both contributed to focused and canalised auxin movement during vascular differentiation; it was proposed that *KAN* genes act by downregulating PIN activity, and that *HDZIPIII*s promote the differentiation of xylem tissues, including the auxin-conducting xylem parenchyma [42]. A relatively subtle change in the HDZIPIII / KAN activity balance characteristic for the r-protein mutants, with lowered HD/ZIPIII or increased KAN activity, might not be critical for bud auxin export and activation in wild type, but might prevent buds of *max2* from activating when there is a higher auxin load in the main stem.

## Conclusions

Our analysis of RPS10B function suggests a role in patterning and in boundary establishment at the shoot apex, processes that are intimately connected with dynamic regulation of auxin flows. Furthermore, RPS10B is required to sustain the outgrowth of *max2* axillary buds in the presence of auxin, while it is largely dispensable for bud outgrowth otherwise. Regulation of development is not likely to be a specialised role of RPS10B within the S10e protein family. However *rps10b-1* and other r-protein mutants highlight the importance of ribosomal function for normal development. Combined with advances in the study of ribosomal activities [122], they might in the future help us to understand how plant ribosomal biogenesis and translation are controlled and integrated with development and growth.

## Methods

### Plants and growth conditions

Ecotype Col-0 was used as the wild-type control, and unless stated otherwise mutant lines were in this genetic background. The following lines were described previously: *amp1-1*[67,69], *axr1-3*[65,123], *brc1-2* and *brc2-1* (SALK_091920 and SALK_023116 [28]), *fhy3-12*[73], *max2-1*[54], *max4-1*[62] and *pid-14*[34,76]. Two lines obtained from T-DNA mutant collections were characterised by sequencing from both T-DNA borders: SALK_102345, an insertion in the last exon of *REV* (At5g60690) upstream of the termination codon, and GABI-KAT line GK_302G09, an insertion affecting the second exon of *CUC3* (At1g76420). Multiple mutants which we constructed were confirmed by genotyping, using wild-type and T-DNA allele-specific PCR for insertional alleles, and CAPS [124] or dCAPS [125] markers for point mutation alleles; except for *max4-1,* where homozygosity was confirmed by testing progeny for uniform BASTA-resistance. As *REV* and *RPS10B* are linked, a reduced frequency of double mutant individuals was expected in the F_2_ of the *rps10b* x *rev* cross. Therefore, 36 *rps10b-1* homozygous F_2_ were selected based on their seedling leaf phenotype, genotyped for *RPS10B* and *REV*, and their leaf and lateral shoot development was observed. For the cross *rps10b* x *pid-14*, genotyping was used in the F_2_ to identify *rps10b-1 pid-14/+* individuals expected to segregate the double mutant in the F_3_, with *RPS10B pid-14/+* individuals used as controls. About 40 F_3_ progeny each were then genotyped and phenotyped.

Arabidopsis seeds were sown onto Levington F2 compost pretreated with systemic insecticide (Intercept 70WG, Everris Limited, Ipswich, UK). Trays were chilled at 4°C for 3 days and then incubated in a greenhouse with 16-h supplemental lighting. These conditions were used for all soil-grown plants except for the hypocotyls examined by histology (Figure 3). These were from 14-day-old plants grown in continuous low light (40 μmoles m^-2^ sec^-1^ from white fluorescent tubes, 21°C) and from 60-day-old plants grown in short (8-h) photoperiods (160 μmoles m^-2^ sec^-1^ from fluorescent white tubes, 21°C day / 17°C night temperature). Except for the mutant screen described below, individual plants were grown at a density of 1 per 16 cm^2^ in trays consisting of 40 x 16 cm^2^ compartments.

### Identification of RPS10B as a *max2-1* suppressor

*max2-1* seeds were mutagenised with 0.3% ethyl methanesulfonate. 18 000 seeds from the resulting M_2_ generation were sown at densities of one plant per 3 or 5 cm^2^ and screened for reduced rosette branching at maturity. One of the suppressor mutations isolated, *6-7*, was recessive and segregated independently from *max2-1* after backcrossing to Columbia wild-type. The suppressor locus was mapped to a 126-kbp interval on Chromosome 5 using about 1600 mutant individuals from the F_2_ of a cross between L*er* plants and the *67* mutant in the *MAX2* background. End-sequenced TAC clones from the Arabidopsis wild-type Columbia genomic JAtY library in pYLTAC17 [57] with inserts spanning the mapping interval were obtained from the John Innes Genome Laboratory, and transformed into Agrobacterium strain GV3101 for floral dipping of *6-7 MAX2*. This was done according to Clough and Bent [126], except that the infiltration medium contained glucose instead of sucrose. T_1_ selected for BASTA-resistance under sterile conditions were further cultivated on soil. Their phenotypic rescue was scored; and they were genotyped to confirm the presence of the left and right vector – genomic insert borders specific to the TAC clone.

### RNA isolation, RT-PCR, cloning

Total RNA was extracted using the RNeasy plant miniprep kit with on-column DNaseI digestion (Qiagen, Hilden, Germany) from about 100 mg tissue powder, obtained from 10 pooled 1-cm primary inflorescence stem base segments per genotype, from bolting plants of about 25 cm height. cDNA synthesis was performed from 1 μg total RNA in a total volume of 10 μl with SuperscriptII (Invitrogen, Life Technologies, Carlsbad, CA) and oligo-dT primer. After diluting each sample by adding 70 μl of water, 2 μl were used in 50 μl semi-quantitative PCR reactions with 26 cycles, unless stated otherwise. Gene-specific *RPS10A-*, *RPS10B-* and *RPS10C* primer pairs were used. RT-PCR for *ACTIN2* (At3g18780) was used as RNA input control. Primer sequences are listed in Table 5.

**Table 5:** Primers used in this study

**Primer name**	**Sequence 5’ → 3’**
RPS10B genomic construct	
RPS10Bgenomic-F	AAACTAGTAACCGAGTAAACGGGATGATTAGG
RPS10Bgenomic-R	AAAAAAAGCTTAGCTCCTCAACATTCAACTCCTTC
RPS10B promoter::RPS10A, RPS10B and RPS10C cDNA constructs	
RPS10Bpro-F	GGATCC GCGGCCGCTGAATAAGTAACATCAAACTCCAGCTA
RPS10Bpro-R	ATCATGATTGCGATGAGATTGAAGAAGGA
RPS10AcDNA-F	CTTCAATCTCATCGCAATCATGATTATCTCAGAGAACAATCGCAG
RPS10AcDNA-R	AATCTAGATCAAGGGAACCCTGAACCAGATGGTGCT
RPS10BcDNA-F	CTTCAATCTCATCGCAATCATGATCATATCAGAGACTAACCGCCGT
RPS10BcDNA-R	AATCTAGA TCAAGGAAGATCAGATCCAGCAGCA
RPS10CcDNA-F	CTTCAATCTCATCGCAATCATGATTATCTCAGAGGCTAACCGCAAA
RPS10CcDNA-R	AATCTAGATCAAGGCAAACCTGAACCAGATGGTGCA
RT-PCR	
RPS10A-RT-F	AGATTTGGTGACCGTGATGGATAC
RPS10A-RT-R	CCTTCCATCGTCGCAATATGAC
RPS10B-RT-F	AGGTTTGGTGACAGAGATGGATAC
RPS10B-RT-R	AGACCAAAAAGAAACAAGAAAGTCC
RPS10C-RT-F	GTTTGGTGACCGTGATGGGTAC
RPS10C-RT-R	AACTCCTCCATGGTCTTACTGTC
CCD7-F	CCGAGTCAAGCTTAATCCAATAG
CCD7-R	ATTGCAGTTTCCGGTAGAGTCCAA
CCD8-F	CATCGGCGATCAACAAATAA
CCD8-R	GTTTAACCAAATCCGGTATC
Ubiquitin5-F	AACCCTTGAGGTTGAATCATC
Ubiquitin5-R	GTCCTTCTTTCTGGTAAACGT
Actin2-RT-F	TTACCCGATGGGCAAGTCA
Actin2-AT-Rev	CACCACTGAGCACAATGTTAC

A genomic *RPS10B* construct was produced by amplifying a 3.5 kb fragment spanning the *RPS10B* genomic region from Columbia wild-type genomic DNA with primers RPS10BgenomicF and RPS10BgenomicR (Table 5). This was digested with SpeI and HindIII and cloned into binary vector pCAMBIA2300 (http://www.cambia.org) opened with XbaI and HindIII for plant transformation.

To express *RPS10A, RPS10B, RPS10C* cDNAs under the *RPS10B* promoter, *RPS10B* promoter region was amplified from Columbia wild-type genomic DNA, and the *RPS10A (*At4g25740.1)*, RPS10B* (At5g41520.1) and *RPS10C* (At5g52650.1) coding regions were amplified from Columbia wild-type cDNA using the primers specified in Table 5. The three forward primers for the *RPS10* coding regions introduced an overlap with the *RPS10B* promoter amplicon, which was then fused upstream of each cDNA by overlap extension in a second round of polymerase chain reaction. Furthermore, the primers introduced a BamHI site followed by a NotI site just upstream of the promoter and an XbaI site just downstream of the termination codon. The products were digested with BamHI and XbaI and ligated into the cloning vector pART7 [127] opened with the same enzymes. Inserts were confirmed by sequencing. From these plasmids, NotI releases a fragment consisting of the *RPS10B* promoter, the *RPS10A*, *B* or *C* coding region and the plasmid-encoded octopine synthase gene terminator, which was transferred into a NotI-digested derivative of the plant transformation vector pART27 [127] which confers BASTA-resistance in plants. Confirmed constructs were shuttled into Agrobacterium strain GV3101 and used for plant transformation [126].

### Auxin physiology and transport, histology of hypocotyl sections stained for DR5::GUS activity

Axillary bud outgrowth assays were performed with cauline nodes excised from the primary inflorescence of plants grown in sterile conditions, as described [59]. 2-^14^C-indoleacetic acid transport assays were conducted with 1.5-cm stem segments from the basal internode of the primary inflorescence of 6-week old soil-grown plants [55,60].

2 mm of apical hypocotyl tissue and the cotyledonary node of 14 day-old seedlings germinated under continuous illumination and 2-mm segments from the thickened hypocotyls of 60-day-old short-day-grown plants were stained for β-glucuronidase (GUS) activity at 37°C overnight, fixed for 5 h, and embedded in Technovit (Heraeus Kulzer, Hanau, Germany); 10 μm transverse sections were prepared, mounted to slides, counterstained with ruthenium red, and permanently mounted as described [119].

## Competing interests

The authors declare no competing interests.

## Authors’ contributions

PS isolated the mutant, performed the genetic and phenotypic characterisation, and made the *RPS10* constructs. JPL, SLK and SW mapped the mutant gene. JPL and PS performed mutant rescue experiments. Excised node assays were carried out by SW. PS and OL wrote the manuscript. All authors read and approved the final manuscript.

## Supplementary Material

Additional file 1 **Table S1. ***rps10b-1 * complementation analysis. Click here for file

Additional file 2 **Figure S1.***rps10b-1 * does not suppress strigolactone insensitivity of * max2-1 *hypocotyls. Relative hypocotyl lengths of light-grown wild-type, * rps10b- *1, * max2-1 * and * rps10b-1 max2-1 * seedlings after 7 days of growth on vertical sterile agar plates without or with the synthetic strigolactone GR24. Mean hypocotyl lengths (n = 19-28), were normalized to the mean length on control medium for each genotype. Error bars represent the standard error of the ratios. Sterile growth conditions and preparation of GR24 according to [55] except that sucrose was omitted from the growth medium. Click here for file

Additional file 3 **Figure S2.***rps10b-1 * does not suppress upregulation of the genes encoding strigolactone biosynthetic enzymes CCD7 (CAROTENOID CLEAVAGE DIOXYGENASE7) and CCD8 in * max2- *1 mutant inflorescence stems. RT-PCR analysis of the transcript levels of * CCD7 * and *CCD8* in total RNA prepared from basal primary inflorescence stem segments. RT-PCR for * UBIQUITIN5 * (*UBQ5*) was used as RNA normalization control. Click here for file

Additional file 4 **Figure S3.** Widespread expression of * RPS10A *, * RPS10B * and *RPS10C and lack of tissue-specific variation in their relative contributions to transcript level. * RT-PCR analysis of the transcript levels of *RPS10A*, * RPS10B * and * RPS10C * in total RNA prepared from different Columbia wild-type Arabidopsis tissues was carried out as described [119]. Gene-specific amplification was ensured by reverse priming to divergent 3’-untranslated sequences. RT-PCR for *ACTIN2* was used as RNA normalization control. Click here for file

Additional file 5 **Figure S4.** rps10b-1 enhances leaf polarity defects of the * asymmetric leaves1 (as1)* mutant. The * as1-1 * allele in the Col-1 background (NASC stock N3374) was used in this experiment. Rosette centres of wild type (**a**), *rps10b-1* (**b**) and *as1* (**c**) controls and of putative double mutant *rps10b-1 as1* F_2_ segregants from a cross of the single mutants (**d**, **e**). While the oldest leaves of these plants appeared *as1*-like, younger leaves were trumpet-shaped, or their leaf lamina was strongly reduced (arrows). These segregants bolted normally and produced flowers and seeds. Scale bars: 5 mm in (**d**) for (**a**-**d**) and 1 mm in (**e**). (PDF 1583 kb) Click here for file
