# Supplementary material for: Mutation of the cytosolic ribosomal protein-encoding RPS10B gene affects shoot meristematic function in Arabidopsis
Source: BMC Plant Biol. 2012 Sep 10;12:160. doi: 10.1186/1471-2229-12-160 (PMC3492191; doi:10.1186/1471-2229-12-160)
Supplement: Additional file 1 — Table S1. rps10b-1 complementation analysis. [file 1471-2229-12-160-S1.pdf]

Table S1 - *rps10b-1* complementation analysis

Construct [a]	Transformant generation scored [c]	% of plants with at least one empty apical rosette leaf axil [d]	cauline leaf axil [d]	n
JAtY55I14 [b]	T_2	94.7	34.2	38
JAtY70G08 [b]	T_2	2.5	0	40
RPS10B genomic	T_1	0	0	44

[a] The *rps10b-1* single mutant was used for floral dipping with the constructs.

[b] The genomic insert of TAC clone JAtY70G08 (but not that of JAtY55I14) spans *RBS10B*.

[c] Transgenic seedlings were selected for their construct-specific antibiotic resistance on sterile plates, transplanted to soil and scored at late reproductive stage.

[d] The apical leaf axils, exposed at the top of the rosette, and all the cauline leaf axils were scored by the naked eye for absence or presence of an axillary shoot.
