# Supplementary figures and images for: Mutation of the cytosolic ribosomal protein-encoding RPS10B gene affects shoot meristematic function in Arabidopsis
Source: BMC Plant Biol. 2012 Sep 10;12:160. doi: 10.1186/1471-2229-12-160 (PMC3492191; doi:10.1186/1471-2229-12-160)

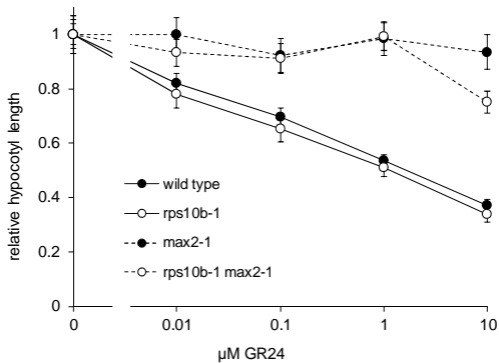

Supplement: Additional file 2 — Figure S1.rps10b-1 does not suppress strigolactone insensitivity of max2-1 hypocotyls. Relative hypocotyl lengths of light-grown wild-type, rps10b- 1, max2-1 and rps10b-1 max2-1 seedlings after 7 days of growth on vertical sterile agar plates without or with the synthetic strigolactone GR24. Mean hypocotyl lengths (n = 19-28), were normalized to the mean length on control medium for each genotype. Error bars represent the standard error of the ratios. Sterile growth conditions and preparation of GR24 according to [55] except that sucrose was omitted from the growth medium. [file 1471-2229-12-160-S2.pdf]

wt

*rps10b-1*

*max2-1*

*rps10b-1 max2-1*

*CCD7*  
35 cyc

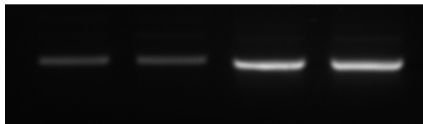

*CCD8*  
35 cyc

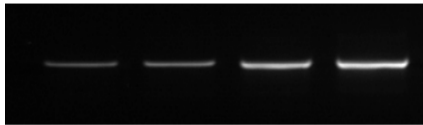

*UBQ5*  
30 cyc

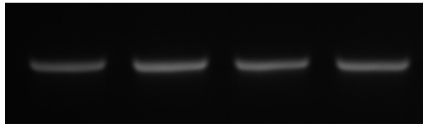

Supplement: Additional file 3 — Figure S2.rps10b-1 does not suppress upregulation of the genes encoding strigolactone biosynthetic enzymes CCD7 (CAROTENOID CLEAVAGE DIOXYGENASE7) and CCD8 in max2- 1 mutant inflorescence stems. RT-PCR analysis of the transcript levels of CCD7 and CCD8 in total RNA prepared from basal primary inflorescence stem segments. RT-PCR for UBIQUITIN5 (UBQ5) was used as RNA normalization control. [file 1471-2229-12-160-S3.pdf]

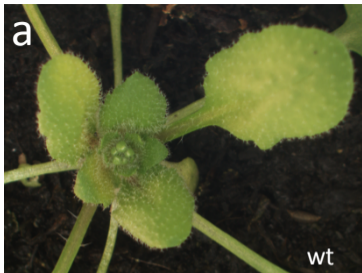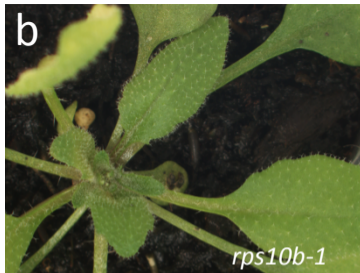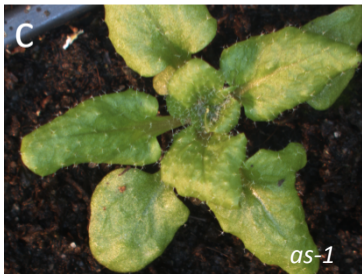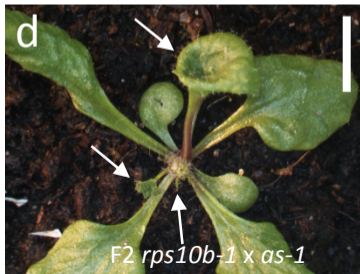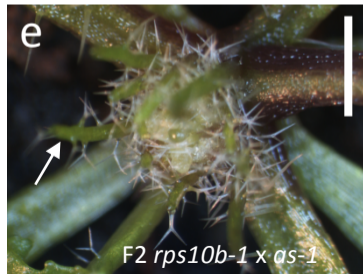

Supplement: Additional file 5 — Figure S4. rps10b-1 enhances leaf polarity defects of the asymmetric leaves1 (as1) mutant. The as1-1 allele in the Col-1 background (NASC stock N3374) was used in this experiment. Rosette centres of wild type (a), rps10b-1 (b) and as1 (c) controls and of putative double mutant rps10b-1 as1 F2 segregants from a cross of the single mutants (d, e). While the oldest leaves of these plants appeared as1-like, younger leaves were trumpet-shaped, or their leaf lamina was strongly reduced (arrows). These segregants bolted normally and produced flowers and seeds. Scale bars: 5 mm in (d) for (a-d) and 1 mm in (e). (PDF 1583 kb) [file 1471-2229-12-160-S5.pdf]
