# Supplementary material for: Mutation of the cytosolic ribosomal protein-encoding RPS10B gene affects shoot meristematic function in Arabidopsis
Source: BMC Plant Biol. 2012 Sep 10;12:160. doi: 10.1186/1471-2229-12-160 (PMC3492191; doi:10.1186/1471-2229-12-160)
Supplement: Additional file 4 — Figure S3. Widespread expression of RPS10A , RPS10B and RPS10C and lack of tissue-specific variation in their relative contributions to transcript level. RT-PCR analysis of the transcript levels of RPS10A, RPS10B and RPS10C in total RNA prepared from different Columbia wild-type Arabidopsis tissues was carried out as described [119]. Gene-specific amplification was ensured by reverse priming to divergent 3’-untranslated sequences. RT-PCR for ACTIN2 was used as RNA normalization control. [file 1471-2229-12-160-S4.pdf]

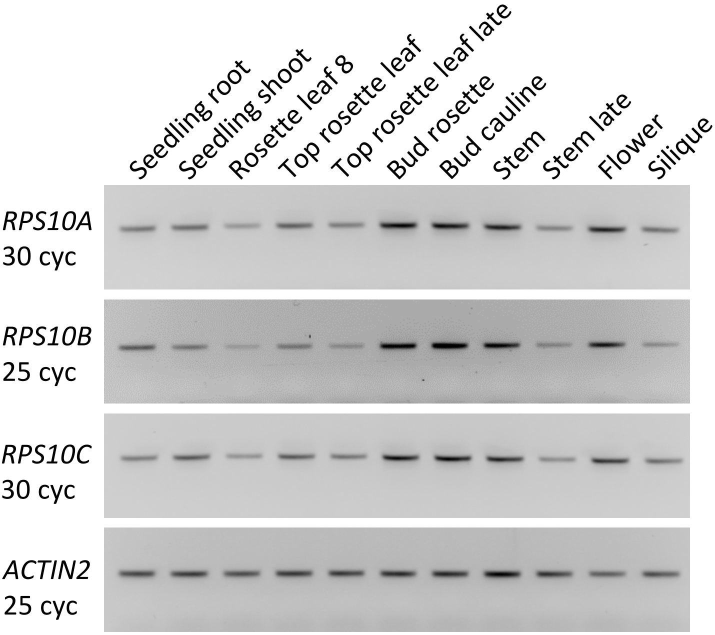
Seedling root
Seedling shoot
Rosette leaf 8
Top rosette leaf
Top rosette leaf late
Bud rosette
Bud cauline
Stem
Stem late
Flower
Silique
RPS10A
30 cyc
RPS10B
25 cyc
RPS10C
30 cyc
ACTIN2
25 cyc
